# Investigation of the effectiveness of sound enrichment in the treatment of tinnitus due to hearing loss

**DOI:** 10.1002/brb3.3520

**Published:** 2024-05-07

**Authors:** Eser Sendesen, Didem Turkyilmaz

**Affiliations:** ^1^ Department of Audiology Hacettepe University Ankara Turkey

**Keywords:** residual inhibition, sound enrichment, tinnitus, tinnitus pitch

## Abstract

**Objective:**

In previous animal studies, sound enhancement reduced tinnitus perception in cases associated with hearing loss. The aim of this study was to investigate the efficacy of sound enrichment therapy in tinnitus treatment by developing a protocol that includes criteria for psychoacoustic characteristics of tinnitus to determine whether the etiology is related to hearing loss.

**Methods:**

A total of 96 patients with chronic tinnitus were included in the study. Fifty‐two patients in the study group and 44 patients in the placebo group considered residual inhibition (RI) outcomes and tinnitus pitches. Both groups received sound enrichment treatment with different spectrum contents. The tinnitus handicap inventory (THI), visual analog scale (VAS), minimum masking level (MML), and tinnitus loudness level (TLL) results were compared before and at 1, 3, and 6 months after treatment.

**Results:**

There was a statistically significant difference between the groups in THI, VAS, MML, and TLL scores from the first month to all months after treatment (*p* < .01). For the study group, there was a statistically significant decrease in THI, VAS, MML, and TLL scores in the first month (*p* < .01). This decrease continued at a statistically significant level in the third month of posttreatment for THI (*p* < .05) and at all months for VAS‐1 (tinnitus severity) (*p* < .05) and VAS‐2 (tinnitus discomfort) (*p* < .05).

**Conclusion:**

In clinical practice, after excluding other factors related to the tinnitus etiology, sound enrichment treatment can be effective in tinnitus cases where RI is positive and the tinnitus pitch is matched with a hearing loss between 45 and 55 dB HL in a relatively short period of 1 month.

## Graphical abstract

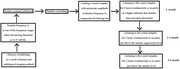

## 1 INTRODUCTION

In adults, about 15% of the population suffers from chronic tinnitus, and about 6%–25% of this group report that it directly affects their daily lives (Eggermont & Roberts, 2004). Tinnitus can cause significant problems such as sleep deprivation (Schecklmann et al., 2015), depression (Langguth et al., 2011), and cognitive problems (Andersson, 2009). To date, the overall goal of tinnitus management has been to reduce the discomfort associated with tinnitus (Jastreboff, 2004). For this purpose, researchers have applied therapy to individuals with chronic tinnitus by using smartphone‐based applications by presenting sounds at a level of intensity that was mixed with tinnitus, regardless of the individual's audiogram (Abouzari et al., 2020; Kutyba, Gos, et al., 2022; Kutyba, Jędrzejczak, et al., 2022; Tyler et al., 2018). The main goal of these studies was to reduce the discomfort caused by tinnitus based on the working principles of the limbic system (Jastreboff, 2004). Although these therapies have been shown to be beneficial, it may be more effective to treat tinnitus as a symptom by directly targeting the tinnitus etiology.

The most common cause of tinnitus appears to be auditory deprivation, as most tinnitus patients also have hearing loss (Langguth et al., 2013). Cochlear damage results in reduced excitatory input to brain regions associated with the frequency range of the hearing loss, as well as reduced inhibition at nearby frequencies (Auerbach et al., 2014; Noreña, 2011). Such a failure of sensory information from the cochlea will result in a compensatory increase in neural amplification, that is, excitation in the central auditory system. This is called “central gain enhancement” and refers to the long‐term alteration of inhibitory synapses at various levels of the auditory system after cochlear damage (Auerbach et al., 2014; Noreña, 2011). Central gain enhancement may initially be distal to the auditory system and then spread proximally (Auerbach et al., 2014). It has been suggested that central gain enhancement is associated with tinnitus perception (De Ridder et al., 2015).

Studies on animals with high‐frequency hearing loss due to noise exposure have shown that keeping these animals in an acoustic environment enriched with high frequencies prevents maladaptive reorganization in the central auditory system (Noreña & Eggermont, 2005). In another study, it was reported that the level of spontaneous activity in the central auditory system was not different from that of their normal hearing peers after high‐frequency sound enrichment was applied to a group of animals with the same condition (Noreña & Eggermont, 2006).

In the literature, there are studies that use sound enrichment to target the area of hearing loss in tinnitus patients (Cuesta & Cobo, 2024; Cuesta et al., 2022; Vanneste et al., 2013). Except for the study by Vanneste et al. (2013), improvement was observed in tinnitus patients in other studies. However, in none of these studies were the results controlled with a placebo group. Moreover, in the abovementioned animal studies, sound enrichment was beneficial by improving the central auditory system only in cases of hearing loss. However, as is known, there are also tinnitus etiology other than hearing loss, such as temporomandibular joint disorder, neck muscle traumas, and metabolic problems (Chan, 2009). In some tinnitus patients whose etiology is not hearing loss, sound enrichment may increase the perception of tinnitus (Kaltenbach, 2006). However, in the study of Vanneste et al. (2013), they did not define a criterion (tinnitus loudness level (TLL), tinnitus pitch, residual inhibition [RI] characteristics, etc.) for associating the etiology of tinnitus patients with hearing loss.

It has been previously stated that tinnitus associated with hearing loss occurs with a spontaneous increase in activity in the central auditory system (Brinkmann et al., 2021). Therefore, RI, which provides clues as to whether the increase in spontaneous activity in the central auditory system is associated with tinnitus, emerges as an important variable in the investigation of the etiology of tinnitus associated with hearing loss (Galazyuk et al., 2019). According to the discordant theory, tinnitus associated with hearing loss occurs at the level where the outer hair cells are most damaged and the inner hair cells are least damaged; that is, the afferent and efferent fibers are most incompatible. This level is approximately 45–55 dB HL in sensorineural hearing loss (Jastreboff, 2011; Lonsbury‐Martin & Martin, 1990; Schaette & Kempter, 2006; Shekhawat et al., 2014). The spontaneous activity of the already less damaged Type I nerve fibers is maximized at this level of hearing loss due to the suppression effect of the almost completely eliminated Type II efferent fibers, and thus tinnitus occurs (Jastreboff, 2011). Therefore, a patient's hearing threshold of 45–55 dB HL at the frequency at which tinnitus occurs may indicate that the etiology of tinnitus may be associated with hearing loss (Jastreboff, 2011; Schaette & Kempter, 2006).

As a result, as shown in animal studies, sound enhancement was beneficial only in patients with tinnitus associated with the etiology of hearing loss. For this reason, it may be important to develop a treatment protocol by considering the factors that will increase the possibility of tinnitus etiology being associated with hearing loss, such as the range of hearing thresholds at the frequency at which RI and tinnitus occur. At the same time, according to the discordant theory, it has been suggested that the increase in spontaneous activity in the central auditory system is maximum at the tinnitus pitch (Jastreboff, 2004; Schaette & Kempter, 2006). In the study of Vanneste et al., this situation was ignored, and the spectrum of the music they used for treatment was created independently of the hearing loss configuration of the patients and without considering the tinnitus pitch.

For these reasons, this study aims to determine criteria to match the etiology of tinnitus patients with hearing loss, to develop a new sound enrichment treatment protocol by producing and applying a sound complex suitable for the hearing loss configuration and considering the tinnitus pitch, and to evaluate the effectiveness of this treatment protocol. We hypothesized that using sound enrichment treatment within the framework of our recommended protocol would improve tinnitus.

## 2 MATERIALS AND METHODS

### 2.1 Participants

Our study includes the data of 127 tinnitus patients who were admitted to our clinic between December 2022 and September 2023 and refused to use hearing aids despite being recommended to them. They were recruited after the following evaluations to include tinnitus patients with hearing loss as the primary etiology. Six participants with potential organic problems (three of these are demyelinating diseases, two are middle‐ear pathologies, and one is a brain lesion) related to the tinnitus etiology were excluded by examining their medical history, radiological images, and audiological evaluation results. External and middle ear functions were normal in the participants’ otoscopic and tympanometric examinations. In patients whose hearing loss configuration showed a decrease toward higher frequencies, the patient was included in the study if the tinnitus pitch was in one of the frequency ranges where the hearing threshold was 45–55 dB HL. If the patient's hearing threshold was outside this threshold range at tinnitus pitch, these patients (11 participants) were excluded from the study because of the possibility that a factor other than hearing loss could be the primary cause of tinnitus (Han et al., 2009; Jastreboff & Hazell, 1993). Moreover, 10 participants with negative RI were excluded. Pure tone audiometry was conducted using an Interacoustics AC‐40 audiometer and calibrated TDH‐39P (0.125–8 kHz) headphones, a Sennheiser HDA200 (9–20 kHz), and a Radioear B‐71 bone vibrator. Four participants with a hearing threshold of more than 90 dB HL in the 0.125–20 kHz range were excluded from the study to benefit from the sound enrichment. All participants also had tonal tinnitus.

As a result, the study included 96 participants with sensorineural hearing loss between the ages of 18 and 43. These participants were randomly assigned to groups by one researcher. The 52 participants in the study group (21 males and 31 females) were diagnosed with chronic tinnitus (more than 6 months) and ranged in age from 18 to 43. The placebo group (19 males and 25 females) consists of 44 participants diagnosed with chronic tinnitus (more than 6 months) and ranges in age from 21 to 42. Tinnitus assessment, tinnitus handicap inventory (THI), and visual analog scale (VAS) scores of the participants were retrospectively analyzed before and during the sound enrichment treatment at all 1, 3, and 6‐month follow‐up visits. All these assessments were made by a blinded researcher who did not know which participant was in which group. Ethical approval for this study obtained from Ethics Committee (SBA23/287).

### 2.2 Tinnitus assessment

TDH‐39P headphones were used to evaluate tinnitus with frequencies below 8 kHz, whereas Sennheiser HDA200 headphones were used to assess tinnitus with frequencies above 8 kHz. In tinnitus pitch matching, a two‐alternative forced selection procedure was used with tonal stimuli presented at 30 dB SL between 0.125 and 20 kHz from the contralateral ear to prevent the patient from becoming confused by tinnitus (Yakunina & Nam, 2021). Considering the participants’ ipsilateral hearing threshold at the tinnitus pitch, the TLL was then matched in 5 dB HL steps (Henry & Meikle, 2000).

The minimum masking level (MML) was found to be the level at which the center of tinnitus pitch was masked by narrowband noise in 5 dB steps (the Interacoustics AC‐40 audiometer allows steps of 5 dB minimum) (Henry & Meikle, 2000). Participants with bilateral or central tinnitus were exposed to narrowband noise bilaterally. It was presented to the tinnitus ear in unilateral tinnitus condition.

Finally, RI was determined by presenting narrowband noise matching the tinnitus perception with a center frequency of 10 dB above the MML for 60 s (presented in the tinnitus ear is unilateral, binaural if tinnitus is bilateral) (Roberts, 2007). These results were considered positive if the tinnitus perception level decreased and negative if the tinnitus did not change or if the tinnitus perception level increased. It is known that after continuous stimulation of a sensory neuron, its spontaneous activity decreases below its initial spontaneous activity (Fournier et al., 2018). If RI is positive, tinnitus decreases as spontaneous activity decreases; therefore, an increase in spontaneous activity may be associated with tinnitus. Therefore, only tinnitus patients with positive RI were included in this study.

### 2.3 Tinnitus questionnaire

We used the THI to measure the impact of tinnitus on the participants’ daily lives (Aksoy et al., 2007). THI contains 25 questions and assesses the subjective psychological effects of tinnitus as reported by patients. Functional, emotional, and destructive subscales associated with tinnitus are assessed using the THI. The answers are “Yes,” “Sometimes,” and “ No.” These answers are evaluated as 4, 2, and 0 points, respectively.

### 2.4 Visual analog scale (VAS)

The VAS was used to measure subjectively perceived tinnitus severity (VAS‐1), discomfort (VAS‐2), attention deficit (VAS‐3), and level of sleep difficulties (VAS‐4) due to tinnitus. The participants were instructed as follows: “Rate the conditions in front of you for yourself on a scale of 0–10. A score of 0 means ‘none,’ while a score of 10 means ‘unbearable.’ Intermediate emotions can be marked somewhere between 0‐10 points.”

### 2.5 Sound enrichment treatment protocol

All patients with positive RI, tinnitus pitch in the range of 45–55 dB HL hearing loss, and meeting both criteria were included in the study.

Counseling was given to tinnitus patients before starting sound enrichment treatment. Peripheral and central auditory systems were explained in basic terms. Neuroplastic changes in the auditory system cause hearing loss, and the mechanisms of tinnitus due to these changes were mentioned. Afterward, information was given about the relationship between tinnitus and the limbic system, which is closely related to emotional reactions. Sound enrichment treatment was started for the patients who completed the counseling session, which lasted approximately 30 min.

**FIGURE 1:**
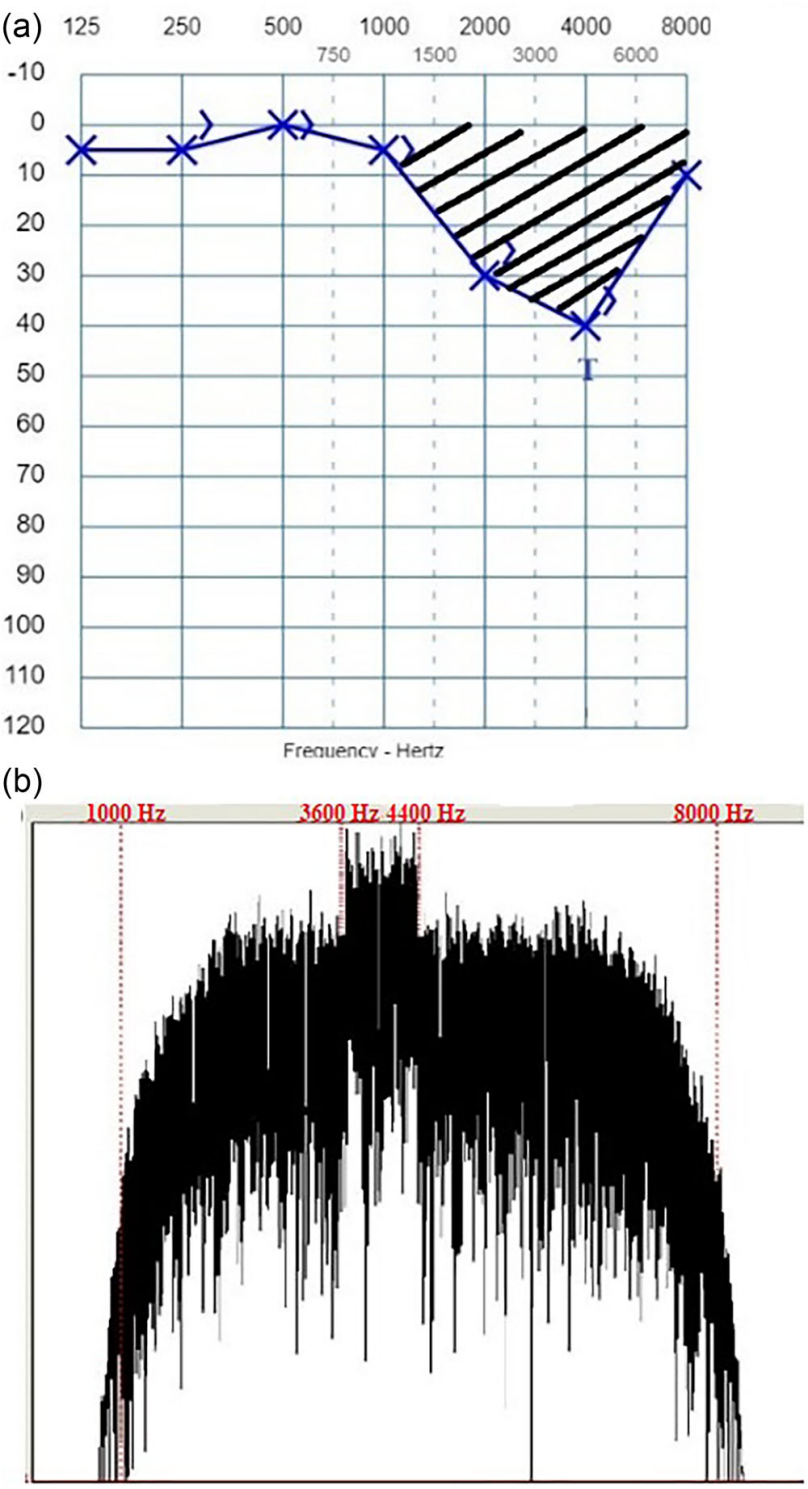
An example audiogram and spectrum of sound complex v3 created for treatment in accordance with the audiogram for the study group. (a) Audiogram of a patient with tinnitus at 4 kHz. The shaded area shows the spectrum range of the sound used for treatment. (b) The spectrum of the sound created for treatment (extra amplification in the range of 3.6–4.4 kHz, in addition to the frequency content in the range of 1–8 kHz).

**FIGURE 2:**
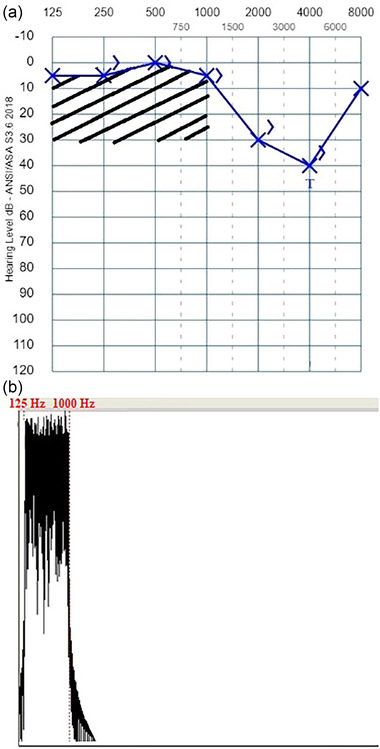
An example audiogram and spectrum of sound complex in accordance with the audiogram for the placebo group. (a) Audiogram of a patient with tinnitus at 4 kHz. The shaded area shows the spectrum range of the sound used for treatment. (b) The spectrum of the sound created for treatment (the frequency content in the range of 0.125–1 kHz).

Based on the audiogram of the patients, it was aimed to create a sound complex using the Praat program; that amplitude envelope is suitable for the hearing loss configuration, contains the frequencies of hearing loss in its spectrum, and this spectrum has the maximum amplitude at the tinnitus pitch. First, white noise with a sampling frequency of 44,100 Hz was generated according to the Gaussian distribution. This white noise was filtered using a band‐pass filter in the patient's hearing loss frequency range. A smoothing parameter was used during the filtering process to ensure that the targeted sound was appropriate for the hearing loss configuration. This parameter was determined according to the difference between the hearing threshold at the frequency where the hearing loss started and the hearing threshold at the adjacent frequency (e.g., if hearing loss starts at 1 kHz, the adjacent frequency is taken as 2 kHz.) with a normal hearing threshold (≤15 dB HL). If this difference was large, a low smoothing was used; if the difference was small, a proportionally high smoothing was used. Finally, a sound complex v1 was created with the spectral content intended to compensate for the hearing loss configuration. Afterward, another white noise was created, and this noise was filtered with a band‐pass filter using a value 10% below and above the patient's determined tinnitus pitch. In this way, the sound complex v2 was created. The primary purpose of designing the sound complex v2 was to reduce the spontaneous activity, which was thought to be maximum at the tinnitus pitch according to the discordant theory, with a sound complex with the maximum amplitude at the tinnitus pitch (Noreña & Eggermont, 2005, 2006). Finally, an individualized sound (sound complex v3) was created by combining sound complex v2 with the sound complex v1 in mono and used for the study group. An example audiogram of the study group and the spectrum of the sound complex v3 based on this audiogram are shown in Figure 1. On the other hand, the sound used in the treatment for the placebo group was created as follows: White noise with a sampling frequency of 44,100 Hz was made according to the Gaussian distribution. Then, this white noise was filtered using a band‐pass filter by selecting the frequency range in which there was no hearing loss. In this group, low smoothing was used to ensure that the spectrum of the sound created was not within the frequencies where hearing loss was present. An example audiogram and spectrum of the sound complex in accordance with the audiogram for the placebo group are shown in Figure 2.

**FIGURE 3:**
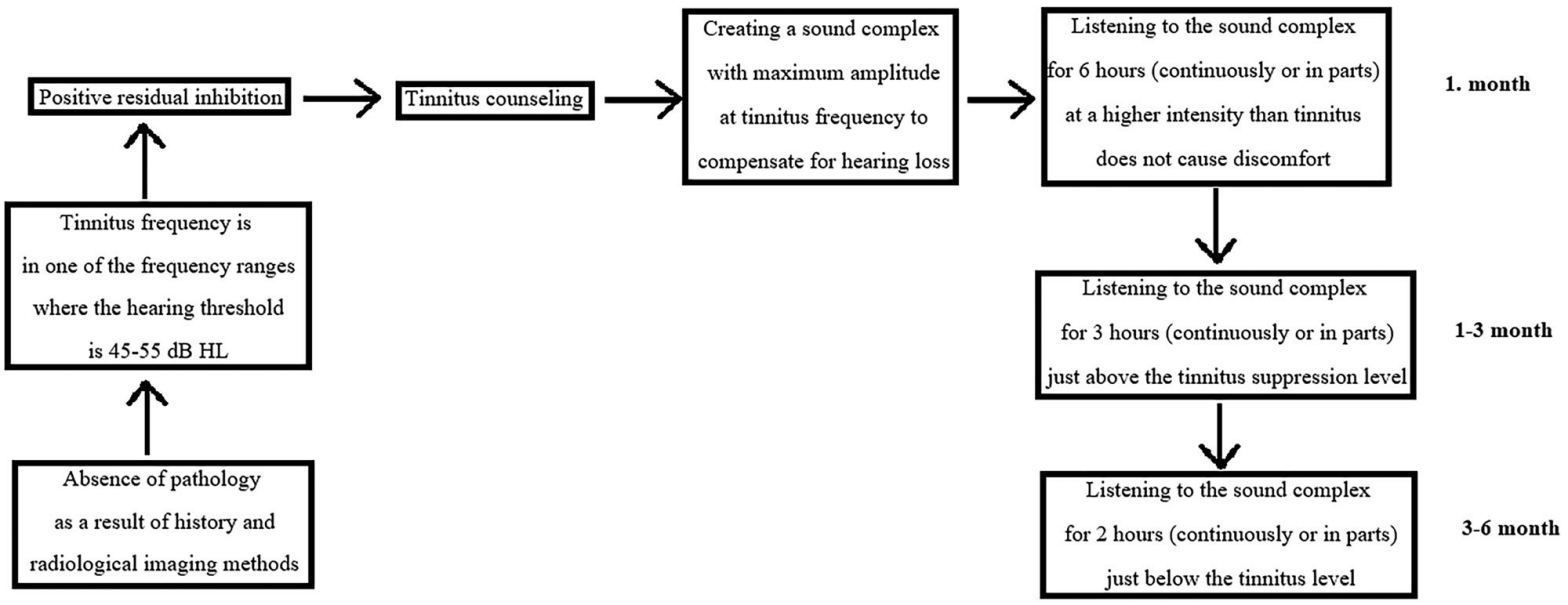
Sound enrichment treatment protocol.

In the first month of the treatment, the individualized sounds created for both groups were requested to be listened to with headphones for a total of 6 h a day, continuously or in parts, at a maximum intensity level higher than the tinnitus intensity and not causing discomfort (e.g., if the participants were uncomfortable on the fifth of the five levels of the volume button on their smartphone, they listened on the fourth level. If they were not disturbed, they listened from the fifth level). They listened to the sounds on their smartphones for cost‐effectiveness and accessibility. Between 1 and 3 months, the total listening time was reduced to 3 h, and the sound intensity was reduced to a level just above the tinnitus suppression level. Between 3 and 6 months, the total listening time was reduced to 2 h, and the sound intensity was reduced to a level just below the tinnitus level. The intensity level of the individualized sounds was explained and showed to the participant prior to treatment, as well as at the first and third‐month follow‐up appointments. For participants whose tinnitus may vary in severity throughout the day, they were instructed to adaptively adjust their individualized sound according to their tinnitus level throughout the day. Moreover, participants do not need to pay attention while listening to their individualized sound. The sound enrichment treatment protocol used in the present study is shown in Figure 3.

The researcher randomly distributed the participants to the groups, designed the sound used for sound enrichment according to the participant's group, and delivered it to the participant. The other researcher applied the sound enrichment treatment protocol without knowing which participant was in which group.

### 2.6 Statistical methods

The G*Power program was used to determine the sample size to be included in the study. Given the mean and standard deviation values obtained from the groups as a result of the pilot study, this study should consist of 16 participants from each study and a placebo group with a 5% type I error level and a 95% minimum power to detect a significant difference. The SPSS version 25.0 (IBM Inc.) package program was used to evaluate the data. All data had a normal distribution. Time‐dependent changes of THI, VAS, TLL, and MML scores within and between groups (study and placebo groups were considered between‐subjects factors) were analyzed using Repeated Measures ANOVA. It was considered statistically significant when the *p* value was <.05.

## 3 RESULTS

### 3.1 Descriptive statistics

**FIGURE 4:**
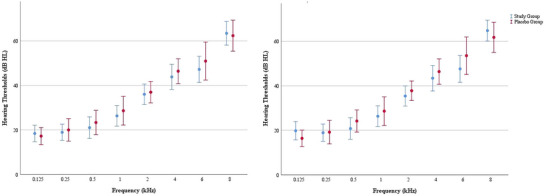
Hearing thresholds of the groups.

Examining the age distribution of the groups reveals that the study group had an average age of 32.21 ± 7.45 years. The mean age of the placebo group was 34.47 ± 7.55 years. Age and sex did not differ significantly between the groups (*p* > .05). General tinnitus characteristics are shown in Table 1. The hearing thresholds of the groups are shown in Figure 4. There was no statistical difference between the groups in terms of hearing thresholds at any frequency (*p* > .05).

**TABLE 1:** General tinnitus characteristics.

Tinnitus location	Study group	Placebo group
Right ear	13	11
Left ear	10	13
In the head/bilateral	29	20
Tinnitus pitch (kHz)	6.2 ± 0.9 (2–8)	6.31 ± 0.7 (1–8)
Tinnitus duration (months)	23.49 ± 10.22 (17–47)	24.68 ± 12.31 (14–52)
Hearing threshold at the tinnitus frequency	51.49 ± 3.14	49.98 ± 4.07

### 3.2 Visual analog scale (VAS)

Table 2 shows the VAS scores of the patients during the treatment and the statistical evaluation between the groups. There was a statistically significant difference between the groups for time‐dependent change of VAS scores (tinnitus severity, *p* < .001, tinnitus discomfort, *p* < .001, attention deficit, *p* < .001, and sleep difficulty, *p* < .001). VAS scores in the study group decreased statistically significantly during the treatment process (tinnitus severity, *p* < .001, tinnitus discomfort, *p* < .001, attention deficit, *p* < .001, and sleep difficulty, *p* < .001), whereas no statistically significant difference was observed in the placebo group (tinnitus severity, *p* = .74, tinnitus discomfort, *p* = .65, attention deficit, *p* = .57, and sleep difficulty, *p* = .63) during the treatment process.

**TABLE 2:** Pre‐ and posttreatment visual analog scale (VAS) scores for the study and placebo groups.

	Study group	Placebo group	Study group	Placebo group	Study group	Placebo group	Study group	Placebo group
	Tinnitus severity		Tinnitus discomfort		Attention deficit		Sleep difficulty	
Time	Mean ± Std.	Mean ± Std.	Mean ± Std.	Mean ± Std.	Mean ± Std.	Mean ± Std.	Mean ± Std.	Mean ± Std.
Pretreatment	7.1 ± 2.2	7.6 ± 1.4	7.2 ± 1.8	7.5 ± 1.6	4 ± 3.1	4.3 ± 2.6	5.6 ± 3.4	6.5 ± 2.7
1 month	4.4 ± 2.4	6.7 ± 1.8	3.5 ± 2.4	6.8 ± 1.7	2.2 ± 2.1	3.7 ± 2.3	2.2 ± 2	5.6 ± 2.5
3 month	2.2 ± 2.4	6.8 ± 1.6	1.8 ± 2.1	6.7 ± 1.2	1.0 ± 0.8	3.2 ± 1.8	1.5 ± 1.8	5.1 ± 2.4
6 month	1.0 ± 1.2	5.8 ± 1.2	1.1 ± 1.1	5.7 ± 1.2	0.7 ± 0.4	3.2 ± 2.1	1.1 ± 1	5.1 ± 2.3
*p* Value*	***<.001***	*.78*	***<.001***	*.84*	***<.001***	*.77*	***<.001***	*.87*

*Note*: Bold *p* values: statistically significant difference.

Abbreviation: Std., standard deviation.

*Repeated measure ANOVA.

### 3.3 Tinnitus handicap inventory (THI)

Table 3 shows the THI scores of the patients during the treatment and the statistical evaluation between the groups. There was a statistically significant difference between the groups for the time‐dependent change of THI scores (*p* < .001). THI scores in the study group decreased statistically significantly during the treatment process (*p* < .001), whereas no statistically significant difference was observed in the placebo group (*p* = .59).

**TABLE 3:** Pre‐ and posttreatment tinnitus loudness level (TLL), minimum masking level (MML), and tinnitus handicap inventory (THI) scores for the study and placebo groups.

	Study group	Placebo group	Study group	Placebo group	Study group	Placebo group
	Tinnitus loudness level		Minimum masking level		Tinnitus handicap inventory	
Time	Mean ± Std.	Mean ± Std.	Mean ± Std.	Mean ± Std.	Mean ± Std.	Mean ± Std.
Pretreatment	61.6 ± 5.8	66.2 ± 17.8	52.7 ± 3.4	55.1 ± 12.8	57.77 ± 25.83	57.58 ± 21.48
1 month	25 ± 10.3	61.2 ± 9.9	16.1 ± 6.9	54.6 ± 11.1	23.33 ± 21.93	56.32 ± 19.96
3 month	19.4 ± 10.5	60.6 ± 11.4	10 ± 6.1	51.2 ± 11.8	12.22 ± 16.53	56.24 ± 19.46
6 month	11.6 ± 6.2	63.1 ± 13.3	6.6 ± 3.9	46.8 ± 8.4	5.77 ± 8.45	54.47 ± 20.66
*p* Value*	***<.001***	*.75*	***<.001***	*.86*	***<.001***	*.66*

*Note*: Bold *p* values: statistically significant difference.

Abbreviation: Std., standard deviation.

*Repeated measure ANOVA.

### 3.4 Tinnitus loudness and minimum masking level

Table 3 shows the TLL and MML scores of the patients during the treatment and the statistical evaluation between the groups. There was a statistically significant difference between the groups for the time‐dependent change of TLL scores (*p* < .001). TLL decreased statistically significantly in the study group during the treatment process (*p* < .001), whereas no statistically significant difference was observed in the placebo group (*p* = .69).

There was a statistically significant difference between the groups for time‐dependent change MML scores (*p* < .001). MML scores decreased statistically significantly in the study group during the treatment process (*p* < .001), whereas no statistically significant difference was observed in the placebo group (*p* = .64).

## 4 DISCUSSION

The study's main aim was to develop a method of sound enrichment that has not been successful in tinnitus suppression before, investigate its effectiveness in existing tinnitus patients, and, if the new method is successful, provide clinical use for patients with tinnitus. According to the present study's results, THI, VAS, TLL, and MML scores improved in the study group compared to the placebo group when sound enrichment treatment was used according to our recommended protocol.

Previous studies have shown that in case of hearing loss, sound enrichment reduces spontaneous activity (Noreña & Eggermont, 2006), causes functional reorganization in the central auditory system (Noreña & Eggermont, 2005), and even plays a protective role in the auditory system by activating the dopaminergic pathway (Niu et al., 2007). It is known that maladaptive reorganization in the central auditory systems of tinnitus patients and increased spontaneous activity compared to healthy individuals have been reported in studies (Isler et al., 2022; Sendesen, Erbil, et al., 2022; Sendesen, Kaynakoglu, et al., 2022). Therefore, as a result of the current study, the improvement seen in tinnitus patients after sound enrichment treatment may be due to the functional reorganization and spontaneous activity reduction caused by sound enrichment. However, it should be kept in mind that it is not possible to indicate this only with the results of subjective evaluation methods such as THI, VAS, TLL, and MML, so the results should be interpreted carefully.

Although sound enrichment is theoretically and according to the present study's results, thought to be beneficial in tinnitus patients, Vanneste et al. (2013) have shown that the application of music modified to have a spectrum that compensates for hearing loss in tinnitus patients does not improve tinnitus and may even provoke tinnitus in some patients. It is known that tinnitus is only a symptom, and it has been shown that it can occur due to several pathologies (Chan, 2009). Therefore, it may be useful to evaluate the tinnitus etiologies before tinnitus treatment because it has been shown that sound enrichment causes spontaneous activity reduction and functional reorganization only in the case of hearing loss (Noreña & Eggermont, 2005). Therefore, sound enrichment treatment can be expected to be effective in tinnitus patients whose etiology is hearing loss. Vanneste et al. did not evaluate the etiology of tinnitus and only included tinnitus patients with hearing loss in their study. However, it is known that hearing loss may not be the primary cause of tinnitus, which occurs in all cases of hearing loss (Coelho et al., 2020; Martines et al., 2010). Some patients may have more than one etiology of tinnitus. For example, the primary tinnitus etiology of a patient with hearing loss may be somatosensory‐based (Levine et al., 2007). It has been shown that somatosensory tinnitus can also be provoked by an external auditory stimulus (Kaltenbach, 2006). Therefore, sound enrichment with music may not have improved tinnitus in Vanneste et al.’s study, as no classification protocol was associated with tinnitus etiology.

There are also studies in the literature that found that sound enrichment improved tinnitus symptoms in tinnitus patients, similar to our findings (Cuesta & Cobo, 2024; Cuesta et al., 2022). However, because these studies did not include a placebo group, it is difficult to determine whether the improvement in tinnitus patients was due to the sound enrichment or the counseling given to participants. Therefore, by using a placebo group with counseling in the present study, we have shown that sound enrichment provides improvement in tinnitus patients. On the other hand, it was observed that the participants’ initial THI scores decreased from 50–60 to 25–35 following treatment in these studies (Cuesta & Cobo, 2024; Cuesta et al., 2022). In the present study, the mean baseline THI scores decreased from 57 in the study group to 5 by the end of the treatment. As these studies did not apply a selection criterion (TLL, tinnitus pitch, RI characteristics, etc.) to the participants in terms of the possibility that their tinnitus could be associated with hearing loss, as did et al.’s studies, there may have been an important distinction in THI scores with our study's results.

While recruiting participants for this study, it was tried to classify the tinnitus etiology and exclude pathologies other than hearing loss as much as possible by medical history and radiological imaging methods. It is also known that tinnitus pitch can give an idea about the etiology of tinnitus (Nicolas‐Puel et al., 2002). According to the discordant theory, tinnitus occurs at the level of hearing loss, at a level of about 50 dB HL, where outer hair cells are most damaged, inner hair cells are least damaged, that is, afferent and efferent fibers are most discordant in the central auditory system (Dubey, 2022; Jastreboff, 2004). Therefore, the present study included patients with tinnitus pitch in regions with a hearing loss level of 45–55 dB HL. It has also been shown that RI is an important assessment tool for determining the relationship between tinnitus and increased spontaneous activity as a result of hearing loss (Galazyuk et al., 2019). A positive RI indicates a decrease in tinnitus after 1 min of narrow‐band noise. It is known that after stimulating a sensory neuron continuously, its spontaneous activity falls below its initial spontaneous activity (Fournier et al., 2018). If RI is positive, tinnitus decreases with the decrease in spontaneous activity; therefore, it can be concluded that the increase in spontaneous activity is potentially associated with tinnitus. Considering that the etiology of hearing loss is directly related to the increase in spontaneous activity in the auditory system (Schaette & Kempter, 2006; Seki & Eggermont, 2003), only tinnitus patients with positive RI were included in the current study. As a result, many factors related to tinnitus etiology were tried to be excluded from the present study. Thus, sound enrichment treatment has been used in cases of tinnitus, which is more likely to be caused by hearing loss and was tried to develop a protocol that would provide maximum improvement in this treatment method.

According to TLL, MML, VAS, and THI results, prominent improvement was observed between pretreatment and posttreatment 1 month compared to other treatment processes. Although tinnitus improvement continued in other months, the most prominent improvement was observed in the first month. In previous studies, sound enrichment was applied 24 h a day to animals after acute acoustic trauma. After 1 month of sound enrichment following acoustic trauma, traces of maladaptive reorganization (such as increased spontaneous activity and damaged tonotopic organization) disappeared in the central auditory system of the animals (Noreña & Eggermont, 2005, 2006). In the current study, after 6 h of sound enrichment treatment per day, a prominent improvement in tinnitus perception in 1 month compared to other treatment processes is consistent with the results of these animal studies.

Although the primary outcome of our study was an improvement in the study group, we believe the placebo group should also be mentioned. Although there was no statistically significant decrease in scores in the placebo group after 6 months, all variables (In TLL, MML, VAS, and THI scores) in the evaluation results showed a numerical decrease. This result may be the effect of counseling given to both groups. Previous studies have shown the effect of counseling given to tinnitus patients on therapy and treatment motilities (Henry et al., 2022; Jastreboff, 2011). Therefore, we think that the effect of counseling in tinnitus management should not be ignored. On the other hand, even though sound enrichment was not applied to the placebo group, the participants thought that they were being treated. Accordingly, the limbic system may have been partially conditioned by the positive reinforcement, thanks to the hope that tinnitus could be improved in the participants, and this may have led to a numerical decrease in the placebo group, although not statistically (Jastreboff, 2004).

The limitations of the study are as follows: Although we have explained the efficacy and basic physiological mechanism of the proposed treatment protocol by combining subjective assessment methods and the results of previous studies, it may be useful to evaluate the effectiveness of this treatment protocol with objective neurophysiological evidence (such as EEG, fMRI, and auditory evoked potentials) in future studies. Furthermore, as part of the sound enrichment treatment, participants were instructed to listen to a custom‐made sound for a predetermined amount of time and intensity. Although participants reported finishing these durations with the specified intensity, there was no actual monitoring of sound enrichment usage time and intensity. Likewise, we confirmed whether the participants received therapy/treatment or counseling elsewhere during the experiment, based on their verbal statements. It would be beneficial for future studies to provide an experimental setup that guarantees these instead of verbal statements. On the other hand, we used the THI in our study because we had access to limited questionnaires in our native language in terms of validity and reliability. It may be useful for future studies to investigate the results with alternative questionnaires.

To the best of our knowledge, this study showed for the first time that after using criteria to question whether the etiology of tinnitus is related to hearing loss, there was a significant improvement in tinnitus perception in only 1 month with sound enrichment treatment in patients who met these criteria. Moreover, it may be important to prove the effectiveness of sound enrichment treatment using a placebo group. This study differs from previous studies that used sound therapy to provide conventional tinnitus habituation by attempting to treat tinnitus by focusing on the etiology of tinnitus. In conclusion, in clinical practice, after excluding other factors related to the tinnitus etiology, sound enrichment treatment can be effective in a relatively short period of 1 month in tinnitus cases where RI is positive and the tinnitus pitch is matched with a level of hearing loss between 45 and 55 dB HL.

## AUTHOR CONTRIBUTIONS

**Eser Sendesen**: Conceptualization; investigation; methodology; visualization; writing—review and editing; formal analysis; data curation; resources; project administration; writing—original draft. **Didem Turkyilmaz**: Visualization; supervision.

## CONFLICT OF INTEREST STATEMENT

The authors declare that they have no conflicts of interest.

### PEER REVIEW

The peer review history for this article is available at https://publons.com/publon/10.1002/brb3.3520.

## INFORMED CONSENT

Informed consent was obtained from all individual participants included in the study.

## Data Availability

The data that support the findings of this study are available from the corresponding author, upon reasonable request
